# Laboratory-Based Surveillance of *Neisseria meningitidis* Isolates from Disease Cases in Latin American and Caribbean Countries, SIREVA II 2006–2010

**DOI:** 10.1371/journal.pone.0044102

**Published:** 2012-08-30

**Authors:** Ana Belén Ibarz-Pavón, Ana Paula Lemos, Maria Cecilia Gorla, Mabel Regueira, SIREVA Working Group, Jean-Marc Gabastou

**Affiliations:** 1 Pan American Health Organization, Washington, D.C., United States of America; 2 Division of Medical Biology, Department of Bacteriology, Instituto Adolfo Lutz, São Paulo, Brazil; 3 Department of Bacteriology, ANLIS-INEI Dr. Carlos G. Malbrán, Buenos Aires, Argentina; University of Ottawa, Canada

## Abstract

**Background:**

Published data on the epidemiology of meningococcal disease in Latin America and the Caribbean region is scarce and, when available, it is often published in Spanish and/or in non-peer-reviewed journals, making it difficult for the international scientific community to have access.

**Methods:**

Laboratory data on 4,735 *Neisseria meningitidis* strains was collected and reported by the National Reference Laboratories in 19 Latin American countries and the Caribbean Epidemiology Centre (CAREC) between 2006 and 2010 as part of the work carried out by the SIREVA II network. Serogroup and MIC to penicillin, rifampin and chloramphenicol were determined.

**Results:**

Isolates were mainly obtained from patients <5 years, but each year around 25% of isolates came from adult patients. Serogroup distribution was highly variable among countries. Serogroup C was the main cause of disease in Brazil; the majority of disease seen in the Southern cone was caused by serogroup B, but serogroup W135 strains have increased in recent years. In the Andean and Mexico, Central America and Caribbean regions, serogroups B and C were equally present, and serogroup Y was frequently isolated. Isolates were generally susceptible to chloramphenicol, penicillin and rifampin, but almost 60% of isolates characterized in Southern cone countries presented intermediate resistance to penicillin. Five rifampin-resistant isolates have been isolated in Uruguay and Brazil.

**Conclusions:**

Serogroup distribution is highly variable among countries, but some geographic structuring can be inferred from these data. Epidemiological and laboratory data are scarce among Andean and Mexico, Central America and Caribbean countries. Evaluation and implementation of corrective measures on disease surveillance and reporting systems and the implementation of molecular diagnostic techniques and molecular characterization on meningococcal isolates are advised.

## Introduction

*Neisseria meningitidis* is a leading cause of invasive disease in the form of meningitis and scepticæmia worldwide. Invasive meningococcal disease (IMD) is associated with a high case-fatality rate and survivors are often left with severe long-term neurological sequelæ [1].

In industrialized countries, IMD is presented in sporadic cases with small localized outbreaks occurring occasionally [2]–[8]. In these countries, incidence rates remain low and rarely surpass 1/100,000 [9]. Large epidemics are restricted to Africa and, specifically to the area known as the “meningitis belt” [10], [11], [12].

Disease causing serogroups vary widely across continents. Serogroup A is associated with epidemic outbreaks in Africa and, occasionally, in other parts of the world, whereas serogroup B is the leading cause of disease in Australia and New Zealand, as well as in Europe [9], [13], especially since the introduction of the meningococcal C conjugate vaccine in the national vaccination schedules of many countries [14]–[17]. In the United States, serogroups B, C and Y account for the majority of disease in the United States, although the implementation of the tetravalent A, C, W135, Y conjugate vaccine for children from the age of eleven has contributed to a substantial reduction on disease incidence [18].

Despite being a disease of compulsory notification and presumably a leading cause of invasive bacterial disease in Latin America, the real burden of meningococcal disease is widely underestimated in the majority of countries in the region. Reported annual incidence rates range from the approximately 2/100,000 cases reported annually from Brazil, a country with a well-established surveillance and reporting system, to figures below 0.1/100,000 in countries such Paraguay, Costa Rica or Mexico, or even countries where no cases of disease have been confirmed for years [19].

The reasons for this are diverse and also vary from country to country, but differences on clinical definition of cases and management of patients, difficulties on the isolation, identification and characterization of the bacterium, and under-notification to the national reporting system are common problems in the region [19], [20].

Since 1993, the Pan-American Health Organization (PAHO) has run a laboratory-based regional surveillance system; SIREVA (*Sistema Regional de Vacunas*) and later SIREVA II (*Sistema de Redes de Vigilancia de Agentes Bacterianos Causantes de Meningitis y Neumonías*) aimed at the collection of relevant laboratory and epidemiological data on the invasive disease-causing bacteria, *Streptococcus pneumoniae*, *Haemophilus influenzae* (1997) and, since 2000, *Neisseria meningitidis* [20]–[23].

Meningococcal vaccines are not available through the public sector in Latin American countries, with two exceptions: Cuba introduced in 1991 the Cuban-developed outer-membrane protein based vaccine VA-MENGOC-BC®, which achieved a significant reduction of IMD incidence rates in the country [24], [25], [26]. And Brazil is currently the only country in Latin America that has introduced a meningococcal serogroup C conjugate vaccine in its national immunization scheme for children under the age of two [27], but in contrast to the majority of European countries, no catch-up campaign on teenagers is planned. However, meningococcal vaccination is available through the private sector in many Latin American countries. It is crucial for public health authorities and policy makers that data regarding distribution and characteristics of disease-causing strains in Latin America is made available so a cost-effective vaccination campaign with the right vaccine formulation can be implemented through the National Program of Immunization, targeting at including all individuals on the selected groups to be vaccinated regardless of their socio-economic status. This paper reviews available laboratory-based data on serogroup distribution and antibiotic resistance of *Neisseria meningitidis* generated by countries from the Latin America and Caribbean region within the SIREVA II network for the period 2006–2010 [28]–[32].

## Materials and Methods

### SIREVA II Network

In 1993, the Pan-American Health Organization (PAHO) started a regional laboratory-based passive surveillance program, aiming at obtaining quality laboratory data on disease-causing *Streptococcus pneumoniae* strains. Surveillance was extended to *Haemophilus influenzae* in 1997 and *Neisseria meningitidis* in 2000, Currently, the SIREVA II network includes 19 national reference laboratories (NRLs) from an equivalent number of countries from the Latin American and Caribbean region participate in the SIREVA II network: Argentina, Bolivia, Brazil, Chile, Colombia, Costa Rica, Cuba, Ecuador, El Salvador, Guatemala, Honduras, México, Nicaragua, Panama, Paraguay, Peru, Dominican Republic, Uruguay and Venezuela. Additionally, the Caribbean Epidemiology Centre (CAREC), which provides reference laboratory services for 21 countries in the Caribbean, is also part of the network. The *Instituto Adolfo Lutz* (IAL) in São Paulo, Brazil serves as the regional reference laboratory (RRL) for *Neisseria meningitidis*, and the Instituto de Salud Carlos III in Madrid, Spain (ISCIII), acts as global reference laboratory for this bacterium [20], [21], [33], [34].

Data have been grouped into four geographic regions: (i) Brazil, (ii) Southern cone (Argentina, Chile, Paraguay and Uruguay), (iii) Andean Region (Bolivia, Colombia, Ecuador, Peru and Venezuela), and (iv) Mexico, Central America and Caribbean (Mexico, CAREC, Costa Rica, Cuba, Dominican Republic, El Salvador, Guatemala, Honduras, Nicaragua, and Panama) (Table 1).

**Table 1:** Isolates received from each participating country.

Country and regions	Year					Total	
	2006	2007	2008	2009	2010		
	n					n	%
**Brazil**	628	563	622	582	686	**3,081**	**65.1**
**South Cone**						**1,126**	**23.8**
Argentina	64	119	142	132	134	591	
Chile	71	82	57	60	56	326	
Paraguay	7	13	13	9	7	49	
Uruguay	40	46	32	22	20	160	
**Andean**						**260**	**5.5**
Colombia	25	39	22	21	17	124	
Venezuela	25	28	24	16	18	111	
Ecuador	6	5	8	0	1	20	
Bolivia	0	0	2	0	0	2	
Peru	0	0	1	1	1	3	
**Mexico, Central America & Caribbean**						**268**	**5.7**
Mexico	10	13	10	2	17	52	
El Salvador	5	6	6	6	2	25	
Nicaragua	2	0	0	2	1	5	
Honduras	1	0	0	2	0	3	
Costa Rica	18	7	7	2	1	35	
Panama	4	9	28	13	12	66	
Dominican Republic	18	10	4	1	10	43	
Cuba	8	4	7	6	6	31	
CAREC	5	3	0	0	0	8	
**TOTAL**	937	947	985	877	989	4735	

### Isolates

Data include a total of 4,735 *Neisseria meningitidis* strains that were received between 2006 and 2010 at the NRLs from the aforementioned countries [28], [29], [30], [31], [32]. Isolates came from national hospitals whose type and distribution varies across countries, but there is a predominance of pædiatric hospitals belonging to the public health sector, although adult population are also attended in many centers and some countries receive isolates also from the private sector. Isolates were obtained from CSF (71.4%), blood (28.0%), and other sterile body fluids (0.6%), and information on gender, age, clinical presentation of the disease, serogroup and antibiotic resistance pattern was available. A total of 3,081 (65.1%) isolates were reported from Brazil alone. Argentina reported 591 (12.5%) isolates, and 326 (6.9%) came from Chile. Uruguay, Colombia and Venezuela reported 160 (3.4%), 124 (2.6%) and 111 (2.3%) respectively. All other countries reported under 100 isolates each (Table 1).

### Laboratory Procedures

*N. meningitidis* isolates received at the NRLs were processed for ascertainment of species identification, serogrouping and determination of Minimum Inhibitory Concentration (MIC) to antibiotics [35]. Determinations were performed by agar dilution when possible [36], or using the gradient diffusion method (*e.g.* Etest, AB Biodisk®) following the manufactureŕs instructions. Results were interpreted according to the MENSURA group [37].

### Quality Assurance

Quality and reliability of data are guaranteed through a comprehensive quality control and assurance assessment that is carried out by the IAL. The program was originally developed for *S. pneumoniae* and later extended to *H. influenzae* and *N. meningitidis*. Twice a year, five meningococcal test strains are distributed among the SIREVA II network participating laboratories, which will perform the necessary tests for species identification, phenotypic characterization (serogroup, serotype and serosubtype) and antibiotic susceptibility testing. A report is issued twice a year with the results for all countries, so corrective measures can be implemented if needed. A similar test is carried out on the IAL by ISCIII. External quality assurance is carried out twice a year, when the NRLs send their strains to the IAL for confirmation [34].

### Statistical Analyses

Proportions were compared using Chi-Square test or Fisheŕs exact tests as appropriate. All analyses were performed using Stata/SE 11 for Windows.

## Results

### Clinical Origin of Isolates

**Figure 1:**
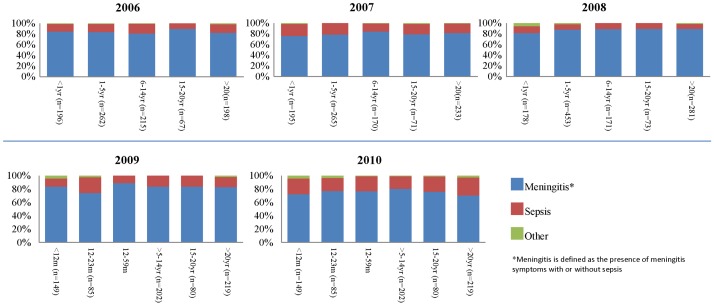
Meningococal disease presentation in Latin America and the Caribbean, 2006–2010. *Meningitis is defined as the presence of meningitis symptoms with or without sepsis.

Around 80% of isolates were obtained from patients with meningitis in all study years (Figure 1). Approximately 20% of isolates came from patients presenting sepsis without meningitis, and around 1% of strains were isolated from cases of forms of invasive disease. Data did not specify what type of disease, with the exception of pneumonia cases, of which there were three in 2006, one in Argentina and two in Ecuador, and another three in 2007: in Chile, Ecuador and Paraguay respectively.

### Isolates Reported by Age Group and Region

Data on age distribution of meningococcal disease isolates is shown in Tables 2 and 3, reflecting changes on the age-distribution data reporting introduced in 2009.

**Table 2:** Age distribution by region, 2006–2008.

Region	2006					2007					2008				
	<1 yr	1–5 yrs	6–14 yrs	15–20 yrs	>20 yrs	<1 yr	1–5 yrs	6–14 yrs	15–20 yrs	>20 yrs	<1 yr	1–5 yrs	6–14 yrs	15–20 yrs	>20 yrs
**Brazil**	98 (15.6%)	194 (30.8%)	153 (24.3%)	53 (8.4%)	132 (21.0%)	95 (16.9%)	164 (29.1%)	121 (21.5%)	43 (7.7%)	140 (24.9%)	79 (12.7%)	178 (28.6%)	121(19.4%)	52 (8.3%)	193 (31.0%)
**Southern cone**	62 (32.4%)	55 (28.8%)	33 (17.3%)	4 (2.1%)	37 (19.4%)	70 (26.6%)	85 (32.3%)	34 (12.9%)	14 (5.4%)	60 (22.8%)	71 (29.5%)	76 (31.5%)	29 (12.0%)	12 (5.0%)	53 (22.0%)
**Andean Region**	19 (35.2%)	6 (11.1%)	6 (11.7%)	6 (11.7%)	17 (31.5%)	24 (33.3%)	10 (13.9%)	11 (15.3%)	7 (9.7%)	20 (27.8%)	13 (22.8%)	10 (17.5%)	9 (15.8%)	2 (3.5%)	23 (40.4%)
**Mexico, Central America & Caribbean**	15 (21.1%)	14 (19.7%)	24 (33.8%)	3 (4.2%)	15 (21.1%)	10 (19.6%)	11 (21.6%)	12 (23.5%)	5 (9.8%)	13 (25.5%)	13 (20.3%)	12 (18.8%)	11(17.2%)	7 (10.9%)	21 (32.8%)
**Total**	194 (20.5%)	269 (28.4%)	216 (22.8%)	66 (7.0%)	201 (21.2%)	199 (21.0%)	270 (28.5%)	178 (18.8%)	69 (7.3%)	233 (24.6%)	176 (17.9%)	276 (28.0%)	170 (17.3%)	73 (7.4%)	290 (29.4%)

**Table 3:** Age distribution by region, 2009–2010.

Region	2009						2010					
	<12 months	12–23 months	24–59 months	≥5–14 years	15–20 years	>20 years	<12 months	12–23 months	24–59 months	≥5–14 years	15–20 years	>20 years
**Brazil**	73 (12.5%)	50 (8.6%)	93 (16.0%)	152 (26.1%)	59 (10.1%)	155 (26.6%)	81 (12.3%)	40 (6.2%)	83 (12.9%)	169 (26.2%)	82 (12.7%)	190 (29.5%)
**Southern cone**	63 (28.3%)	30 (13.5%)	27 (12.1%)	41 (18.4%)	11 (4.9%)	51 (22.9%)	69 (32.4%)	19 (9.1%)	27 (12.7%)	41 (19.2%)	9 (4.2%)	48 (22.5%)
**Andean Region**	12 (31.6)	2 (5.3%)	2 (5.3%)	5 (13.2%)	5 (13.2%)	12 (31.6)	10 (27.0%)	0	8 (21.6%)	10 (27.0%)	3 (8.1%)	6 (16.2%)
**Mexico, Central America & Caribbean**	7 (20.6%)	4 (11.8%)	2 (5.9%)	6 (17.6%)	11 (32.4%)	4 (11.8%)	8 (16.3%)	3 (6.1%)	10 (20.4%)	12 (24.5%)	5 (10.2%)	11 (22.4%)
**Total**	155 (17.7%)	86 (9.8%)	124 (14.1%)	204 (23.3%)	86 (9.8%)	222 (25.3%)	168 (17.8%)	62 (6.6%)	128 (13.6%)	232 (24.6%)	99 (10.5%)	255 (27.0%)

From 2006 to 2008, isolates obtained from cases on patients aged up to 5 years were consistently the most frequent on the five-year period in Brazil and in the Southern cone region. Brazilian isolates obtained from patients <1 year represented 12.7%, and in the Southern cone isolates from patients <1 year surpassed 30% in most years. A total of 185 (29.4%) isolates were obtained from patients aged 15 and over in Brazil during 2006, and that percentage increased steadily over the following years amidst an outbreak of serogroup C disease in recent years affecting older-age groups in some states in the country. In the Southern cone region, the yearly percentages of isolates obtained from this age-group remained stable.

In the Andean region, the highest percentage of isolates obtained from patients below the age of 5 was seen in 2007, with a total of 34 (47.2%) and 33.3% of them belonging to patients <1 year. Isolates on patients aged 15 and over represented 37.5% of the total in the same year.

In the Mexico, Central America and Caribbean region the percentage of cases among patients aged 5 or less were 40.8%, 34.7% and 38.7% in 2006, 2007 and 2008 respectively, with 21.1%, 16.3% and 21% of cases occurring among those <1 year.

The same trends were observed in all four regions for the period 2009–2010 (Table 3), with a high percentage of isolates coming from older patients in Brazil, and most isolates being obtained from young children, and particularly those <12 months in the Southern cone, Andean and Mexico, Central America & CAREC regions.

### Serogroup Distribution by Regions and Countries

**Figure 2:**
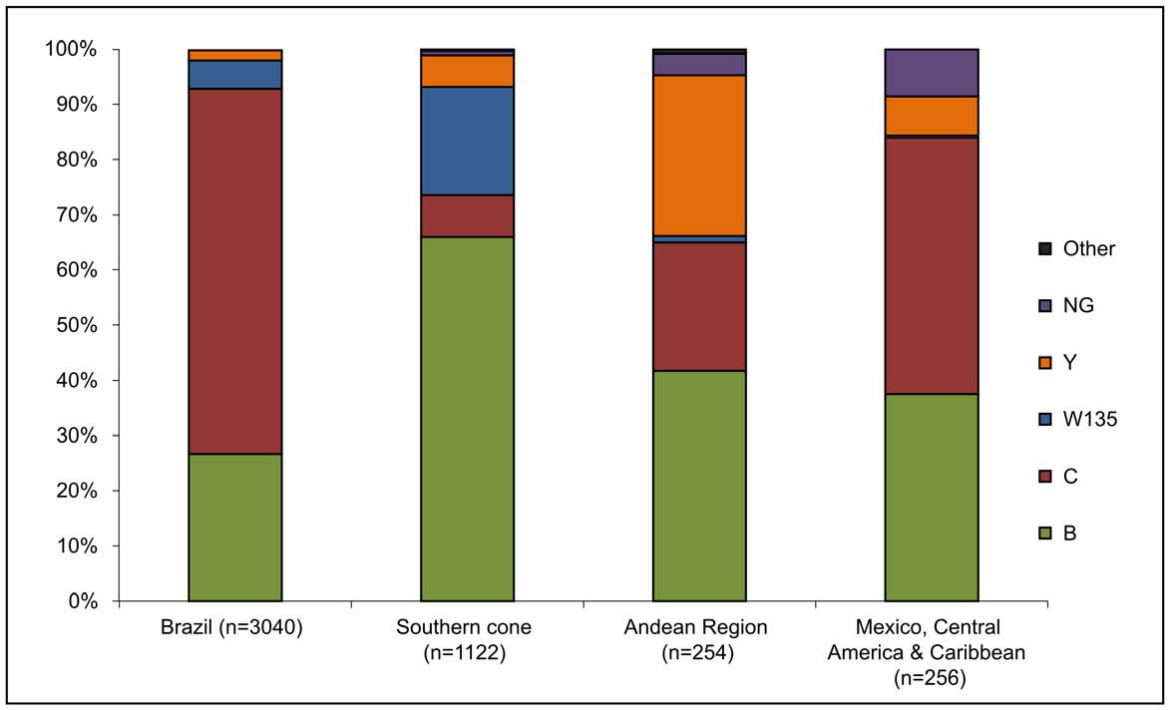
Serogroup distribution, by region for the 2006–2010 period.

Serogroup distribution (Figure 2) was highly variable among countries for the period 2006–2010, which is consistent with what was seen in previous years since *Neisseria meningitidis* surveillance began in 2000 [20]. Sixty-six percent of meningococcal isolates characterized at the Brazil NLR for the period 2006–2010 belonged to serogroup C, and 26.7% were serogroup B. Serogroups W135 and Y represented 5.2 and 1.9% of isolates respectively. Conversely, 66% of all isolates characterized at the NRLs in the Southern cone region belonged to serogroup B, and 7.6% were serogroup C. Serogroup W135 was detected in 19.6% of isolates, and serogroup Y represented 5.8%.

In the Andean region, serogroups B and Y were the most predominant, representing 41.7% and 29.1% respectively. Serogroup W135 accounted for 1.2% of isolates and 23.3% were serogroup C. Serogroup C was the most predominant serogroup among countries located in the Mexico, Central America & Caribbean region (46.5% of all isolates). Serogroup B was found in 37.5% of the isolates, and serogroups W135 and Y were 0.4% and 7% respectively.

**Figure 3:**
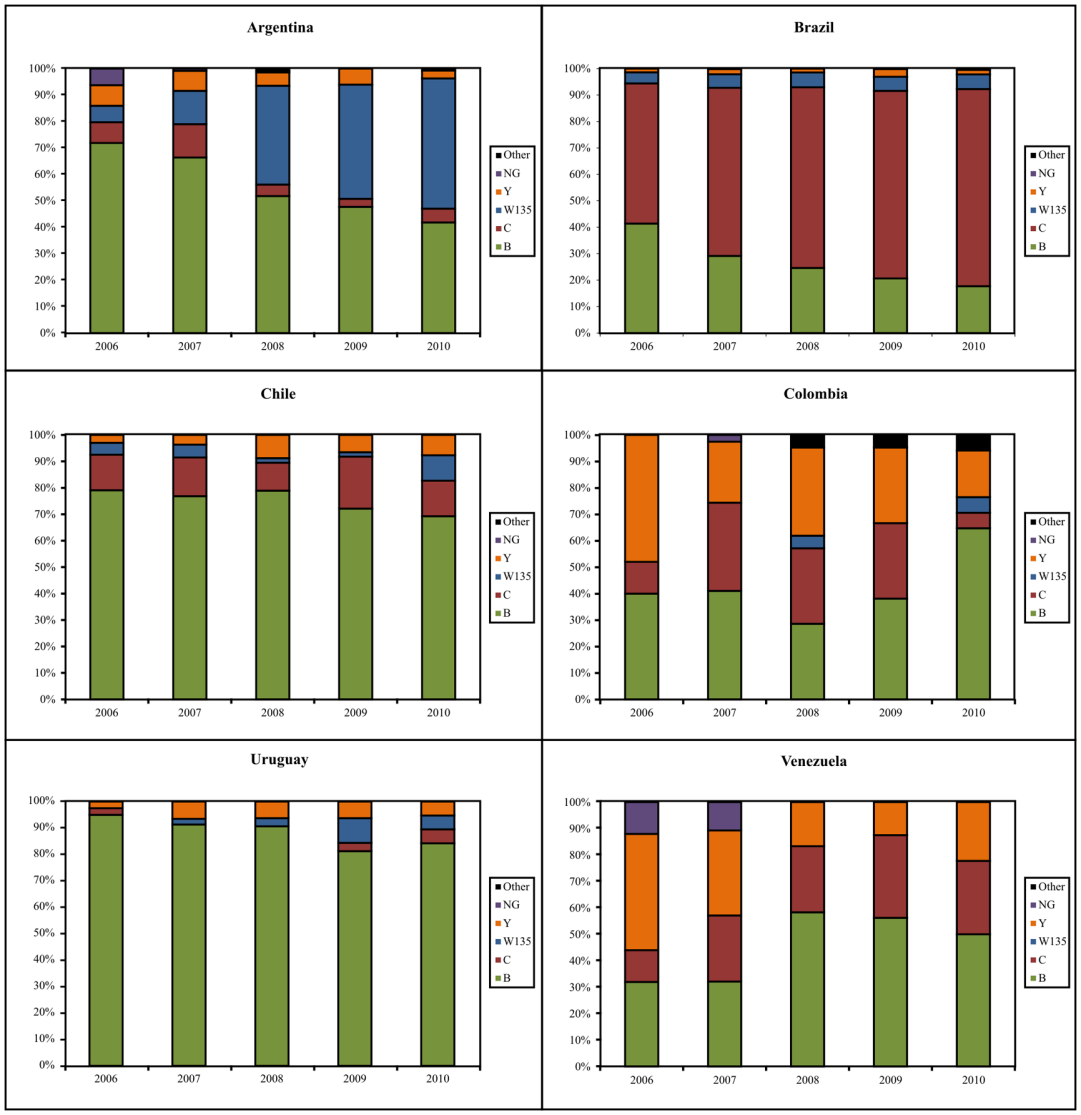
Serogroup distribution by year for Argentina Brazil, Chile, Uruguay and Venezuela. *Selected countries are those for which >100 isolates were available.

Figure 3 shows serogroup distribution among those countries that have reported at least 100 meningococcal isolates for the study period. In Argentina, serogroup B isolates represented 71.9% of all characterized isolates in 2006, whereas these isolates made up to 41.8% in 2010 (p>0.0001). Similarly, serogroup C halved its prevalence, from 7.8% in 2006 to 3% in 2009, but it was higher again in 2010, when it was found in 5.22% of isolates (p = 0.018). The biggest change was noticed in the proportion of isolates belonging to serogroup W135, which represented 6.3% of isolates in 2006 but accounted for almost half the isolates received by the NRL from 2008 onwards.

In Brazil, serogroup C has experienced an important and progressive increase since the year 2000 [20], [28], [30], [31], [38] and accounted for over 74% of isolates characterized 2010 (p<0.0001). Conversely, serogroup B disease represented over 70% of isolates in 2000, but it was only found in 17.7% of them in 2010 (p<0.0001). Serogroups W135 and Y remained more or less stable since 2006 at around 5% and 2% respectively.

Serogroup B has been consistently the causative serogroup of over 80% of cases in Chile since data reporting to SIREVA II began in 2000. Serogroup C remains the second cause of disease and represents between 10–15% of the total. Serogroup W135 accounted for 9.6% of all isolates received in 2010, and serogroup Y isolates doubled their prevalence during the study period. None of these changes were statistically significant.

Serogroup B was present in over 90% of isolates received at the Uruguayan NRL consistently for the entire study period, and other serogroups were rare. In Colombia, serogroup B isolates were the most frequently received by the NRL. An important increase in serogroup Y has been noticed and reported in recent years but our data show that this trend is not sustained over time [39].

Serogroup W135 disease has not been detected in Venezuela, but serogroup Y isolates have been received occasionally by the NRL since surveillance started in 2000 [20]. The percentage of cases caused by this serogroup peaked in 2006, accounting for 44% of the total isolates characterized. Serogroup B isolates accounted for 32% of all meningococcal isolates processed at the NRL in 2006 and 2007, but it became over 50% on the three following years. Serogroup C isolates, however, remained at around 25% of isolates over the 5 years period.

### Serogroup Distribution by Age Group

Serogroup distribution by age for the period 2006–2010 can be seen in Tables 4 and 5. Serogroup B isolates were consistently the most frequently obtained among those that came from patients aged >5 years in the LAC region. Serogroup C isolates, however appeared to be more prevalent among those coming from older patients and, particularly, among teenagers and adults. Similarly, serogroups W135 and Y were also associated with older age, but from 2008 onwards the number of W135 isolates obtained from young children was noticeably higher.

**Table 4:** Serogroup distribution by age, 2006–2008.

Age	2006						2007						2008					
	B	C	W135	Y	NG	Other	B	C	W135	Y	NG	Other*	B	C	W135	Y	NG	Other
**<1**	91 (48.4%)	72 (38.3%)	7 (3.7%)	11(5.9%)	7 (3.7%)	0	107 (65.2%)	27 (16.5%)	14 (8.5%)	14 (8.5%)	2 (1.2%)	0	90 (51.4%)	56 (32%)	22 (12.6%)	6 (3.4%)	0	1(0.6%)
**1–5**	125 (49.8%)	102 (40.6%)	12 (4.8%)	7 (2.8%)	5 (2.0%)	0	126 (46.7%)	119 (44.1%)	13 (4.8%)	6 (2.2%)	6 (2.2%)	0	103 (37.8%)	135 (49.4%)	24 (8.8%)	8 (2.9%)	3 (1.1%)	0
**6–14**	101(47.4%)	95 (44.6%)	5 (2.3%)	7 (3.3%)	5 (2.3%)	0	67 (37.9%)	92 (51.9%)	5 (2.8%)	8 (4.5%)	5 (2.8%)	0	56 (33.1%)	92 (54.4%)	12 (7.1%)	9 (5.3%)	0	0
**15–20**	28 (42.4%)	27 (40.9%)	4 (6.1%)	7 (10.6%)	0	0	22 (32.8%)	35 (52.2%)	3 (4.5)	7 (10.4%)	0	0	23 (29.15)	42 (53.2%)	6 (7.6%)	5 (6.3%)	3 (3.8%)	0
**>20**	96 (47.8%)	80 (39.8%)	7 (3.5%)	17 (8.4%)	1 (0.5%)	0	76 (32.9%)	119 (51.5%)	15 (6.5%)	19 (8.2%)	1(0.4%)	1 (0.4%)	71 (25.4%)	160 (57.1%)	29 (10.4%)	19 (6.8%)	0	1 (0.4%)
**Total**	540	376	37	45	19	0	398	392	442	54	14	1	343	485	93	39	6	2

**Table 5:** Serogroup distribution by age, 2009–2010.

Age	2009						2010					
	B	C	W135	Y	NG	Other*	B	C	W135	Y	NG	Other*
**<12 months**	70 (45.2%)	50 (32.3%)	27 (17.4%)	7 (4.5%)	0	1(0.6%)	62 (36.9%)	61 (36.3%)	40 (23.9%)	5 (3.0%)	0	0
**12–23 months**	37(43.0%)	36 (41.9%)	9 (10.5%)	4 (4.7%)	0	0	23 (37.1%)	30 (48.4%)	5 (8.1%)	4 (6.5%)	0	0
**24–59 months**	48 (39.0%)	68 (55.3%)	5 (4.5%)	2 (1.6%)	0	0	52 (40.6%)	68 (53.1%)	4 (3.1%)	2 (1.6%)	1 (0.8%)	1 (0.8%)
**≥5–14 years**	57 (27,8%)	127 (62.0%)	16 (7.8%)	5 (2.4%)	0	0	64 (27.6%)	145 (62.5%)	18 (7.9%)	4 (1.7%)	1 (0.4%)	0
**15–20 years**	19 (24.1%)	47 (59.5%)	9 (11.4%)	4 (5.1%)	0	0	15 (15.2%)	70 (70.7%)	11 (11.1%)	3 (3.0%)	0	0
**>20 years**	50 (22.0%)	132 (58.1%)	27 (11.9%)	17 (7.5%)	0	1 (0.4%)	51 (20.0%)	155 (60.8%)	32 (12.5%)	12 (4.7%)	1 (0.4%)	4 (1.6%)
**Total**	281	460	93	39	0	2	267	529	110	30	3	5

Serogroup B isolates lowered from 53.1% of all processed isolates in 2006, to 28.3% in 2010 (p<0.0001). Serogroup C isolates varied over the five-years period, but continued to represent around 50% of all those received by the NRLs. Serogroup W135 was found in 3.6% of all isolates received by the NRLs isolates in 2006, but it represented a much higher proportion in 2007, when it was present in 34% of isolates. In 2010, serogroup W135 was detected in 11.7% of isolates in 2010 (p<0.0001).

Data on serogroup distribution by age and regions is submitted as supplementary data (Tables S1 and S2). A decrease in serogroup B across all age groups was noticed in Brazil during the 2006–2008 period, which was accompanied by an increase in serogroup C that was specially marked among older patients. Serogroup C became less prevalent among isolates obtained in the Southern cone region. This serogroup remained generally very low among patients in the <1 year and 1–5 year groups (4–6%), and was more frequent isolated among older patients, representing around 15% of isolates obtained from those aged 6–14, 15–20 and >20 years.

An increase on the percentage of isolates belonging to serogroup W135 was noticed in the Southern cone region across all age groups. In the Andean region, serogroup B represented 16.7% of isolates obtained from those aged 6–14 years in 2006, but it became 44.4% in 2008. An increase on serogroup Y isolates was seen among all age groups.

For the period 2009–2010, serogroup B remained stable in Brazil in all age-groups with the exception of the 15–20 years, among whom changed from 23.7% to 13.4%. Serogroup C went from 62.0% to 70.0% among those aged 12–23 months, and from 67.8% to 79.3% among the 15–20 years group. In the Southern cone region, the proportion of serogroup B isolates among those aged <12 months was 60.3% in 2009, and 47.8% in 2010. Among those in the 24–59 months group, the percentages in 2009 and 2010 were 81.5% and 66.7% respectively, and, 37.7% and 45.7% among the >20 years. Serogroup W135 was found to increase from 2009 to 2010 among adults >20 years, from 29.4% in 2009 to 41.3% in 2010.

### Antibiotic Susceptibility

Minimum inhibitory concentration to penicillin, rifampin and chloramphenicol were determined either at the NRLs of participating countries, or at the RRLs in Brazil. Overall, 70.3% of all isolates were fully susceptible to penicillin, and 29.1% presented an intermediate resistance to this drug. A small decrease in the percentage of isolates with intermediate resistance to penicillin was noticed over the five-year period, as well as in comparison to published data from 2000–2005 [20]. Five fully-resistant isolates were found in 2006: four in Uruguay and one in Mexico. The 14 penicillin-resistant isolates reported in 2010 were found in Mexico, and all but one were isolated from cases of meningococcal disease occurred in penitentiary enclosure.

Isolates remained highly susceptible to rifampin. Intermediately-resistant isolates were rare (0.2%), and only 2 resistant isolates were detected: one in Brazil in 2006 and one in Argentina in 2009. Two rifampin resistant isolates reported in 2010 were isolated in Uruguay. Clinical and epidemiological data has been reported elsewhere [40].

Almost all isolates tested were resistant to chloramphenicol (data not shown) One isolate from Ecuador presented intermediate resistance to this drug, and one fully-resistant isolate was detected in Brazil in 2009.

Tables 6, 7, 8 and 9 show antibiotic resistance data by geographic regions. In Brazil, 14.5% of isolates characterized between 2006–2010 presented intermediate resistance to penicillin, and no fully resistant isolates were found. Over 99% of isolates were susceptible to rifampin, and intermediate and resistant isolates remained rare in Brazil.

**Table 6:** Susceptibility to penicillin and rifampin by year-Brazil.

Year	PENICILIN			RIFAMPICIN		
	Susceptible	Intermediate	Resistant	Susceptible	Intermediate	Resistant
**2006**	168 (80.8%)	40 (19.2%)	0	207 (99.5%)	0	1(0.5%)
**2007**	293 (89.9%)	33 (10.1%)	0	326 (100%)	0	0
**2008**	432 (84.4%)	80 (15.65)	0	511 (100%)	0	0
**2009**	368 (83.6%)	72 (16.4%)	0	440 (100%)	0	0
**2010**	442 (87.4%)	64 (12.6%)	0	502 (99.2%)	3 (0.6%)	1 (0.2%)
**Total**	1703 (85.5%)	289 (14.45%)	0	1986 (99.7%)	3 (0.2%)	2 (0.1%)

* Brazil tests a random selection of isolates (67% in this study). Calculations take into account the percentage of penicillin intermediate-resistance as on the previous year.

**Table 7:** Susceptibility to penicillin and rifampin by year-Southern cone.

Year	PENICILIN			RIFAMPICIN		
	Susceptible	Intermediate	Resistant	Susceptible	Intermediate	Resistant
**2006**	49 (27.8%)	123 (69.9%)	4 (2.3%)	176 (100%)	0	0
**2007**	71 (28.9%)	175 (71.1%)	0	237 (99.6%)	1 (0.4%)	0
**2008**	100 (42.0%)	137 (57.6%)	1 (0.4%)	238 (100%)	0	0
**2009**	110 (51.6%)	103 (48.4%)	0	204 (99.5%)	0	1 (0.5%)
**2010**	108 (50.7%)	105 (49.3%)	0	204 (99.0%)	0	2 (1.0%)
**Total**	438 (40.3%)	643 (59.2%)	5 (0.5%)	1059 (99.6%)	1 (0.1%)	3 (0.3%)

**Table 8:** Susceptibility to penicillin and rifampin by year-Andean region.

Year	PENICILIN			RIFAMPICIN		
	Susceptible	Intermediate	Resistant	Susceptible	Intermediate	Resistant
**2006**	52 (92.9%)	4 (7.1%)	0	55 (98.2%)	1 (1.8%)	0
**2007**	36 (92.3%)	3 (7.7%)	0	39 (100%)	0	0
**2008**	26 (81.3%)	5 (15.6%)	1 (3.1%)	31 (100%)	0	0
**2009**	27 (75.0%)	9 (25.0%)	0	35 (100%)	0	0
**2010**	19 (63.3%)	10 (33.3%)	1 (3.4%)	30 (100%)	0	0
**Total**	160 (82.9%)	31(16.1%)	2 (1.0%)	190 (99.5%)	1 (0.5%)	

**Table 9:** Susceptibility to penicillin and rifampin by year-Mexico, Central America & Caribbean.

Year	PENICILIN			RIFAMPICIN		
	Susceptible	Intermediate	Resistant	Susceptible	Intermediate	Resistant
**2006**	32 (68.1%)	14 (29.8%)	1 (2.1%)	22 (100%)	0	0
**2007**	22 (68.8%)	10 (31.2%)	0	17 (100%)	0	0
**2008**	40 (87.0%)	6 (13.0%)	0	36 (100%)	0	0
**2009**	13 (76.5%)	4 (23.5%)	0	11 (84.6%)	2 (15.4%)	0
**2010**	16 (43.2%)	6 (16.2%)	15 (40.6%)	27 (100%)	0	0
**Total**	123 (68.7%)	40 (22.3%)	16 (8.9%)	113 (98.3%)	2 (1.7%)	

A total of 643 (59.2%) of isolates characterized in the Southern cone region presented intermediate resistance to penicillin, and fully-resistant isolates were also present (four in Uruguay in 2006 and one in Paraguay in 2008) although their overall prevalence was below 1%. Isolates from this region remained susceptible to rifampin, with intermediate and fully resistant isolates being representing 0.1 and 0.3% of isolates respectively.

The Andean Region presented similar percentages of susceptibility to those seen in Brazil. However, the percentage of isolates presenting intermediate resistance to penicillin increased noticeably from 2008 onwards.

In the Mexico, Central America and CAREC, an overall of 68.7% of isolates were fully susceptible to penicillin, and 22.3% presented intermediate resistance to this antibiotic. In 2010, 14 fully penicillin resistant isolates were found in Mexico, all associated to an outbreak of disease in a prison. With the exception of these isolates, penicillin resistance also remained rare in this region.

## Discussion

*Neisseria meningitidis* is presumed to be a leading cause of invasive bacterial disease in Latin America, as the implementation of the Hib-conjugate vaccine has substantially reduced the burden of disease caused by this bacterium [19], [41]–[44]. But to date, very few countries in the region have a well-established surveillance for IMD so no data on the true burden of disease is available. All available laboratory data has been generated through the SIREVA II network, which is coordinated by PAHO [20], [28], [30], [31], [38]. Despite many improvements over the years, quality of both epidemiological and laboratory information is not equal among all participating countries. Specifically, the recovery of *Neisseria meningitidis* is particularly challenging for a number of reasons; empiric antibiotic therapy is often administered prior to sample collection, which prevents the isolation of the bacterium in culture. Delays in sample processing sometimes result in the bacterium being detected by indirect methods such as Gram-stain or latex-agglutination, but not recovered in culture. In many low-income countries, peripheral laboratories lack the infrastructure and resources to allow them to process samples correctly, which results in bacteria being neither detected nor isolated from biological samples. Data presented here was obtained solely from information on the number of strains processed by NRLs and reported to the SIREVA II network, so with a few exceptions, they are not representative of the epidemiology and strain characteristics of IMD in individual countries, but show trends of what has taken place in the region over the past five years. It is worth pointing out that countries such as Argentina, Brazil or Chile, with well-established surveillance system, report to receive isolates from approximately 50 to 60% of all reported cases any given year, whereas Uruguay, which also has a good performing surveillance receives nearly all isolates obtained in the country. But countries such as those located in the Mexico, Central America and Caribbean region and in the Andean region are unlikely to receive any percentage of isolates that could suffice to draw any conclusions regarding the epidemiology or circulating strains in these countries.

Based on the data collected by the NRL in Latin America, IMD in appears to occur mainly in children under the age of 5. However, a substantial number of isolates processed by the NRLs participating in the SIREVA II network were obtained from patients over 20 years of age. Although this could indicate that the number of disease cases among adult patients is unusually high, especially as this trend appears to be sustained over time and also evidenced in countries where clinical management guidelines are well established, such as Brazil or Argentina, further investigations are needed to determine whether and which operational factors could account for this phenomenon in each of the countries. Additionally, the implementation of an active surveillance in a well-defined population including both pædiatric and adults hospitals is needed to confirm this trend. Carriage studies in the region could be of use in providing indirect evidence. In high and medium-income countries, asymptomatic carriage of *Neisseria meningitidis* peaks among adolescents and decreases progressively in adulthood, making disease less likely [45], [46], [47]. Data on the distribution of IMD from Latin America and Caribbean countries could suggests distribution of carriage rates across age groups might differ from those reported from Europe and the US, at least in those where environmental and socio-economic factors make living conditions not comparable to those in other continents. A study performed in Uruguay in 1998 found carriage rates of 41.5% among adults aged 21–30 years, and 19.5% on the 31–40 years age range, which differ from findings elsewhere [48]. However, studies from Cuba [49] and Brazil have shown trends and carriage rates similar to those reported elsewhere [47], [50], [51]. It would therefore be advised that asymptomatic carriage studies are carried out in the region before any vaccination strategy is considered, as the adult group might need to be taken into consideration in some countries if transmission of invasive strains is to be stopped by, for instance, the implementation of a catch-up campaign similar to that carried out in the United Kingdom in addition to the introduction of the vaccine in the national immunization scheme [52].

Serogroup distribution varied widely among the four regions in which countries were divided. Serogroup C was the major cause of disease in Brazil, where a sustained epidemic of serogroup C disease in some states prompted the health authorities to introduce the serogroup C conjugate vaccine in the infant vaccination schedule in October 2010, while changes induced by this public health intervention are being monitored closely [19]. As serogroup C accounted for over 90% of all vaccine-preventable isolates, it is expected that the implementation of the vaccine will substantially reduce the overall burden of disease in the country. In the Southern cone region serogroup C isolates represented less than 10% of those processed by the NRLs, with serogroup B being the major cause of MD. A substantial increase of serogroup W135 was noticed among countries in this region in recent years, especially in Argentina. Brazil reported also an increase on the number of cases of W135 disease in two states: Rio de Janeiro and Rio Grande do Sul. In both cases, the causing strain was found to be that linked to the 2000 Hajj global outbreak [53], [54]. Data presented in this article appear to indicate that serogroup W135 is more often seen among isolates obtained from older patients. It is widely accepted that an age-shift towards older patients occurs in hyper-endemic and epidemic situations [55], [56], which can occur when a new strain is introduced into the population as no immunity has been generated against it [57], [58].

Data on serogroup distribution from the Andean Region and from the Mexico, Central America and CAREC is not representative of the regions, as virtually all data comes from Colombia and Venezuela in the first case and from Mexico, Dominican Republic and Panama on the second case. This was expected as some of the lowest income countries in Latin America are grouped within these two regions.

Analysis of data from Argentina, Brazil, Chile, Colombia, Uruguay and Venezuela highlight variations in the epidemiology of the disease among individual countries. Although all four of the major disease-causing serogroups, B, C, W135 and Y are present across the region, data suggest that recommendations regarding public health interventions to control IMD will need to be carefully evaluated in each country. However, countries grouped in the Southern cone region (Argentina, Chile and Uruguay) all present a similar pattern of serogroup distribution, with high prevalence of serogroup B disease, low number of serogroups C and a recent increase in W135 isolates. Similarly, Colombia and Venezuela, which are both grouped in the Andean region, also share the pattern, with serogroups B and C being both a major cause of disease, and high percentages of serogroup Y compared to other countries in the region. This serogroup was reported to have increased significantly in Colombia since 2003 onwards [39] but our data suggest that this trend might be reverting. A previously published study on the molecular characteristics of serogroup Y isolates found in Latin America showed that the strains circulating in Colombia are not different from those found in Argentina, Brazil, Chile or Costa Rica and hence this apparently unusual situation could not be attributed to the presence of a particularly virulent strain in Colombia [59]. Our data does not suggest that serogroup Y is replacing any of the previously circulating serogroups but rather that there is a higher circulation of this serogroup among the Colombian and Venezuelan populations. Serogroup Y strains are also present in Ecuador, also grouped in the Andean region, and its presence in other surrounding countries could not be ruled out. Close monitoring of disease-causing strains, and particularly of serogroup Y in all countries in the Andean region should be maintained.

Although the number of isolates analyzed from countries grouped in the Mexico, Central America and Caribbean region is not enough to discern a common pattern of major disease-causing serogroups, based on results from the other two major regions, data suggest that some geographic structuring is present among the four regions in which the data has been grouped. This finding could have implications to those countries experiencing difficulties to isolate meningococcus in their laboratories, as they could infer the epidemiology of meningococcal disease based on findings from neighboring countries, notwithstanding the necessity of taking steps to improve their surveillance systems and laboratory capacities to generate their own data.

The number of isolates reported from many of the countries has experienced an important decline, especially when all data collected on the 2000–2005 period are taken into consideration [20]. Evidence points at this decline being caused by the cyclical nature of meningococcal disease outbreaks, which is characterized by waves of progressive increased incidence rates followed by an equally progressive decrease that can last for decades [18], [60], [61].

In many Latin American countries a decline on disease incidence rates that is consistent with this observation has also been noticed [62]–[65]. One explanation for this could be the widespread practice of administering antibiotics prior to LP, which is very common in the region and results in IMD cases being reported as unknown ætiology when reported at all. However, the cyclical nature of meningococcal disease could also provide an explanation for this and if so, increases in the number of isolates reported through the SIREVA II network as well as in disease incidence are to be expected in the future.

All *Neisseria meningitidis* strains for which antibiotic susceptibility was tested remained highly sensitive to rifampin and chloramphenicol. However, the presence of sporadic cases caused by rifampin-resistant strains evidences the need to maintain systematic monitoring of resistance to this drug, as it is still the first choice as prophylaxis for case contacts in many countries in the region.

Decreased sensitivity to penicillin was found in around 30% of isolates. Special attention should be paid in the Southern cone region, where isolates presenting intermediate resistance to penicillin represented almost 60% of all characterized isolates. A number of studies have shown that the infection with a such strains is not associated with a fatal outcome [66], [67], [68], as the drug remains effective for the treatment of IMD. But as the opposite has also been documented, it would be advisable that attention is paid by NRL and epidemiologist to the outcome of IMD cases from whom a strain with decreased susceptibility to penicillin is isolated in order to establish the clinical significance of such strains in the region. These results also highlight the necessity to maintain systematic surveillance of MICs to this drug, as well as the importance of standardizing protocols and unifying susceptibility reporting criteria so ensure data are comparable among countries in the region.

Fourteen penicillin-resistant isolates reported in 2010 belong to an outbreak occurred in a Mexican prison. The outbreak affected mainly inmates and some of their relatives, but at least one unrelated case of penicillin-resistant meningococcal disease was reported (Monica Viveros-Terrazas, personal communication). These results need to be considered with caution as confirmation from the RRL is still pending, but attention must be paid should they be confirmed, as the spread of fully-resistant strains in a region where penicillin is the first line of action when a case of acute bacterial meningitis is suspected could have fatal consequences for the patients.

In conclusion, laboratory data on meningococcal disease across the Latin American region evidences that disease surveillance needs to be improved, especially among countries located in the Mexico, Central America and Caribbean, and those in the Andean region. Information is urgently needed, as the use of either the already available meningococcal vaccines against serogroups A, C, W135 and Y or those that are to come covering serogroup B are likely to have a great impact in the epidemiology of IMD in the region.

The establishment of standardized case definitions and clear guidelines on when lumbar puncture or blood culture should be performed would ensure that cases of meningococcal disease are diagnosed, especially since new molecular diagnostic techniques are being incorporated into routine surveillance and making it possible to identify the causative pathogen even when very little quantities of bacterial DNA are present in the sample [69]. Epidemiological surveillance and report systems need to be carefully evaluated to ensure weaknesses at all levels are identified and addressed. It is of great importance that national health authorities adopt these guidelines into their national ones, and accept the need to obtain data of IMD in their countries if improvements are to be made. An active sentinel-site surveillance covering all age-groups and allowing the incorporation of population-based data would be strongly recommended, especially for those countries where IMD is rarely reported.

Harmonization of laboratory procedures is essential not only to ensure data are comparable among countries, but also to facilitate the identification and solution of any methodological problems that might arise. Peripheral laboratories need to be well equipped and supplied with all necessary reagents and quality controlled-culture media to ensure isolation of bacteria. Personnel in these laboratories should be enrolled in periodic trainings carried out at central level by the NRLs to ensure they can perform all laboratory procedures correctly. Additionally, NRLs need to ensure their staff are properly trained and encouraged to implement new emerging technologies. Implementation of molecular characterization techniques such as Multilocus Sequence Typing (MLST) as a routine in NRLs and RRLs would make data from Latin America comparable to that generated and published worldwide.

With the predicted arrival of new meningococcal vaccines and their potential introduction in Latin American countries, the implementation of measures to allow the generation of reliable data on disease burden and circulating strains in those countries where no information is available will be a major challenge in the upcoming years.

## Supporting Information

Table S1 **Serogroup distribution by age and regions 2006–2008.**
(XLSX)

Table S2 **Serogroup distribution by age and regions 2009–2010.**
(XLSX)
